# Biomarker Turnaround Times and Impact on Treatment Decisions in Patients with Advanced Non-Small Cell Lung Carcinoma at a Large Canadian Community Hospital with an Affiliated Regional Cancer Centre

**DOI:** 10.3390/curroncol31030115

**Published:** 2024-03-14

**Authors:** Katelyn E. Fleming, Ava Hupel, Hamid Mithoowani, Tea Lulic-Kuryllo, Mario Valdes

**Affiliations:** 1 Office of Innovation & Research, Grand River Hospital, Kitchener, ON N2G 1G3, Canada; 2 Department of Oncology, London Health Sciences Centre, London, ON N6A 5W9, Canada; 3 Department of Oncology, Grand River Regional Cancer Centre, Kitchener, ON N2G 1G3, Canada

**Keywords:** lung cancer, NSCLC, molecular testing, IHC, NGS

## Abstract

**Background**: Timely reporting of molecular biomarkers is critical in guiding optimal treatment decisions in patients with advanced non-small cell lung carcinoma (NSCLC). Any delays along the tissue or treatment pathway may be associated with suboptimal treatment/outcomes and a reduced quality of life. For many centres, biomarkers are tested off-site. **Methods:** A retrospective chart review of 123 patients with advanced NSCLC seen between 1 June 2021 and 30 June 2022 was conducted. With a focus on core biomarkers (PD L1, EGFR, and ALK), the outcome variables were as follows: total turnaround time (total TAT), divided into pre-laboratory, laboratory, and post-laboratory time intervals, as well as time to treatment decision (TOTD) and time to optimal systemic therapy decision (TOTSD). **Results:** At first consult, only 20.3% of patients had all core biomarker results available. The median total TAT was significantly longer for non-squamous (non-SCC) than squamous cell carcinoma (SCC) specimens (36.5 versus 22 days, *p* < 0.001). The median pre-laboratory time for the entire cohort was 5 calendar days. The median laboratory testing time was greater for non-SCC compared to the SCC specimens (23 versus 12 days, *p* < 0.001). The median time from consult to TOTD was 19 calendar days for the entire cohort. **Conclusions:** This study emphasizes the need for the expansion of regional resources to meet the clinical needs of advanced NSCLC patients treated at a regional cancer centre which uses an off-site molecular laboratory.

## 1. Introduction

Lung cancer is the leading cause of cancer death in Canada [1]. Most patients present in the advanced stage where the goals are to prolong life and palliate symptoms. Optimal first-line therapy in the advanced stage is individualized based on the presence or absence of a biomarker such as a driver mutation for targeted therapy, or the expression of programmed death 1 ligand (PD-L1) for immunotherapy [2,3,4]. In appropriate patients, treatment with targeted therapy or immunotherapy improves survival and quality of life compared to standard chemotherapy [3]. PD-L1 expression should be interpreted in the context of other biomarkers, given overlap with actionable genomic targets.

Biomarker-directed therapy has a significant impact on the prognosis of patients with metastatic NSCLC. Patients with an EGFR mutation who received targeted therapy as opposed to non-targeted therapy had an improvement in overall survival by 7.8 months [5]. As systemic treatment evolved over time, reflecting the decreased rates of first-line chemotherapy and the introduction of reflex molecular testing, ICI, and targeted therapies, survival outcomes also significantly improved [6].

The timely reporting of all core biomarkers is therefore crucial and endorsed by provincial and national guidelines. According to the Ontario Provincial Lung Cancer Tissue Pathway Map [7], biomarker results should be reported within 14 calendar days of receiving a sample at a molecular reference laboratory. Similarly, the College of American Pathologists guidelines on biomarker testing established a benchmark of 10 working days to report biomarker results after sample receipt [8,9].

**Figure 1:**
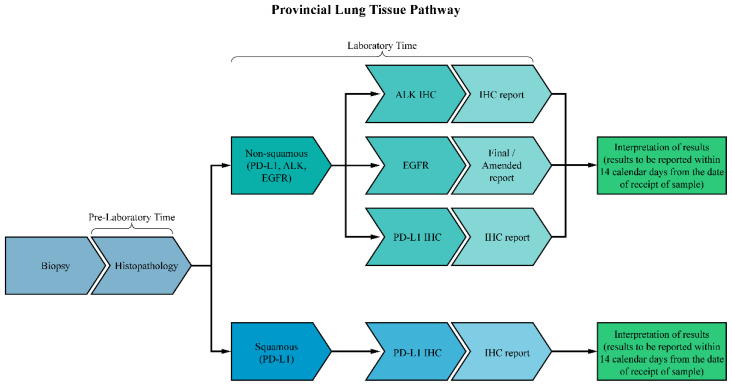
Flowchart depicting the provincial lung tissue pathway. According to the provincial guidelines, all biomarker results should be reported within 14 calendar days for result interpretation.

Additional time is required for processing and shipping biopsy samples if a reference laboratory is not on-site, as is the case across many centres in Canada. The total turnaround time (total TAT) incorporates this by including the time when the histopathologic diagnosis of lung cancer is assigned to arrival at a reference laboratory, as well as subsequent biomarker testing completion. This total TAT should not exceed 21 calendar days [3]. The optimal biomarker testing pathway is shown in Figure 1, which divides the total TAT into the pre-laboratory time (defined as the time from diagnosis to tissue arrival at a reference laboratory), laboratory time (biomarker testing time), and post-laboratory processing (summary report is received by the ordering physician) [10,11]. Separating the total TAT into these phases allows for an analysis of where delays specifically occur and the identification of potential quality improvement.

Delays have previously been reported in centres across Canada, some citing a total turnaround time of up to 6 weeks when a reference laboratory was not on-site. Different reasons have been identified including delays in specimen transportation, insufficient tissue to complete testing, organizational constraints, and volume [3,4,12,13,14,15,16,17]. Although delays in total TAT were defined and quantified in the literature previously, it is not well understood which factors have the greatest impact on prolongation and thus which processes need the most urgent attention to achieve optimization. It has also not been reported whether suboptimal total TAT is associated with a certain histologic subtype.

The goal of this study was to identify and measure delays at each time point within the lung tissue pathway to evaluate the tissue transit, biomarker completion time, and availability of biomarkers in comparison to accepted provincial and national targets. In addition to measuring the testing time in each phase, this study explores how delays in biomarker testing may affect optimal treatment selection in advanced NSCLC. This study was conducted at a large community hospital with an affiliated regional cancer centre where all tissue samples were sent reflexively to an off-site designated reference laboratory for molecular analysis as per provincial guidelines. 

## 2. Materials and Methods

### 2.1. Participants

A retrospective chart review was conducted of newly diagnosed patients with advanced NSCLC treated at a regional cancer centre affiliated with a large community hospital from 1 June 2021 to 30 June 2022. Eligible patients were those ≥18 years of age with biopsy-proven stage IIIb/c NSCLC not amenable to curative intent chemo-radiation, or stage IV NSCLC, with biopsy specimens sent to the designated molecular reference laboratory (MRL). Patients treated in the curative setting were excluded as the role of biomarkers for neoadjuvant and adjuvant therapy was not yet established during the time period of interest. The study was conducted according to the World Medical Association Declaration of Helsinki and approved by the Waterloo-Wellington Research Ethics Board with a waiver of consent (WWREB 2023-0757). 

### 2.2. Data Collection and Analysis

Patient demographics, tumour stage, pathology subtype, comorbidities, performance status at MO consult, and smoking history were extracted from the patient’s electronic medical record (EMR). The molecular analysis is typically composed of immunohistochemistry (IHC) and next-generation sequencing (NGS), whereby IHC results are reported in a partial report and NGS results are supplemented to complete the final/amended report. Both non-SCC and SCC patients receive a partial report, which contains information on PD-L1 and ALK status, while only non-SCC patients receive the final/amended report, which contains information on the core biomarker EGFR. This is the reason why reporting time is typically reduced for SCC patients compared to non-SCC patients. 

**Figure 2:**
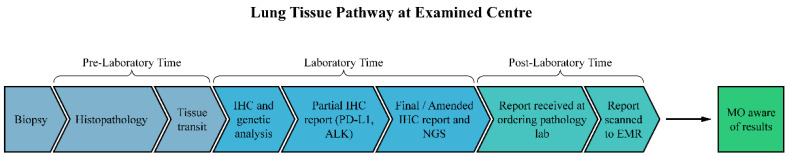
Flowchart depicting the lung tissue pathway at the examined centre. The lung tissue pathway is divided into pre-laboratory, laboratory, and post-laboratory time intervals. The pre-laboratory time interval for a centre employing off-site testing comprises tissue preparation, bar-coding, packaging, and ground transportation to the assigned reference centre. The reference centre for this community hospital completes immunohistochemistry (IHC) on the specimen and prepares tissue for transit to a second laboratory where NGS will take place. Laboratory time thus comprises time to complete both IHC analyses for PDL-1 and ALK mutation and separate genetic analyses (EGFR, etc.). For each analysis, a report is generated (partial versus final/amended) and then faxed to the originating pathology lab at the large community hospital. Post-laboratory time consists of a partial and/or final/amended report received at the ordering pathology laboratory following which it is scanned into the Electronic Medical Record (EMR) system. The consulting medical oncologist (MO) accesses the partial/final (amended) report only when it has been scanned into the EMR.

Time was measured in calendar days at each part of the tissue pathway (Figure 2). Total TAT was defined as the time in calendar days from histopathology to the final molecular report available in the EMR [3]. Total TAT was reported separately by histology. To calculate the pre-laboratory time, the number of days was measured between the date of histopathology and the sample arriving at the molecular reference laboratory (MRL). If there was insufficient tissue for testing, pre-laboratory time was measured from the first sufficient histopathology to tissue arrival at MRL. Laboratory time was measured as the time from receiving the sample at the MRL to the time that the pathologist at the MRL issued a final report. Post-laboratory time was measured as the time from the partial and/or final report MRL sign-off to the report being available locally in the EMR. IHC/NGS results that were not available in the EMR at the time of the retrospective chart review were omitted from the determination of total TAT analysis. Data were also collected on the number of patients receiving empiric treatment without full molecular results, the prescription of radiation while awaiting results, the number of patients with and without results at the time of referral and consult, and the number of patients who were hospitalized or died before an optimal (systemic) treatment decision was made. 

Several other outcome measures related to treatment were examined. Time to optimal treatment decision (TOTD) was measured from the time of the initial medical oncology consultation to a definitive treatment decision (best supportive care or systemic therapy). An optimal treatment decision was based on all biomarkers being available, or optimal treatment could also be the best supportive care irrespective of whether biomarkers were completed. To clarify, for a patient unfit to receive systemic therapy, or a patient who clarified their preference to avoid systemic therapy, TOTD could be captured even if full biomarkers were not available. Additionally, the time to optimal systemic therapy decision (TOTSD) was evaluated. TOTSD was measured from the time of initial medical oncology consultation to a systemic therapy decision and was based on all core biomarkers being available. This outcome included patients being prescribed systemic therapy and excluded patients designated to receive the best supportive care. 

### 2.3. Statistical Analyses

Pre-laboratory, laboratory, and post-laboratory time intervals were described using median and interquartile range (IQR). Differences between groups were examined using the Mann–Whitney U test. Differences within a group were compared using the Wilcoxon sum-rank test. All statistical analyses were performed in MATLAB (Mathworks, Inc., Portola Valley, CA, USA). Statistical significance was set to *p* < 0.05. 

## 3. Results

### 3.1. Patient Demographics, Tumour Characteristics, and Details on Molecular Biomarkers

**Figure 3:**
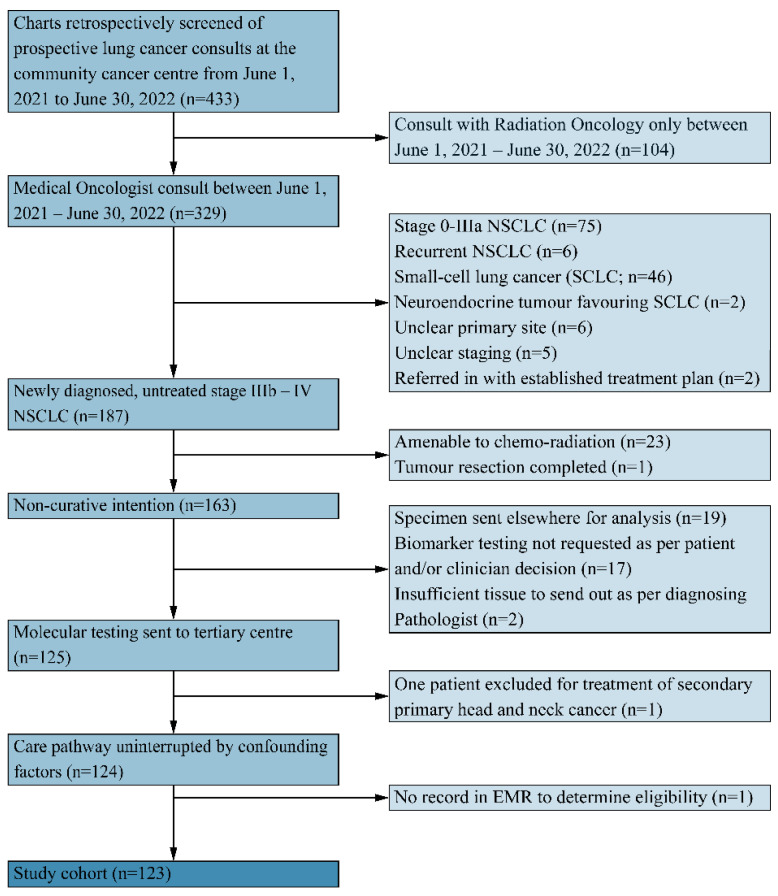
Flowchart depicting patient screening for eligibility in the study.

Out of 433 screened patients, 123 patients met the inclusion criteria as outlined in Figure 3. The patient demographics are summarized in Table 1. The median age was 70.7 years (range: 46–96 years) and 48.8% were male. Adenocarcinoma (non-SCC) was the most common pathologic subtype (73.1%) followed by SCC (22%), undefined pathology sub-type (3.3%), and neuroendocrine/spindle cell carcinoma (1.6%). At the time of the medical oncology consult, seventy-seven non-SCC (85.5%) and sixteen SCC (59.3%) patients did not have all core biomarker results available (Table 2). 

The frequency of EGFR mutations detected across non-SCC patients was 13.3%, and ALK mutations were detected in 1.1% of patients (Table 3). In the total cohort of specimens (both non-SCC and SCC), 48% of patients were defined as PD-L1 positive, 19.5% were PD-L1 low positive, and 23.6% were PD-L1 negative. 

#### Quality of Biopsied Samples and Other Testing 

One or more additional biopsies were required in 17% of patients due to insufficient tissue or low tissue cellularity (Table 2). Seven patients consented to a repeat biopsy. Twelve patients did not have a repeat biopsy for reasons including patient death, deterioration in health, patient preference, alternative testing performed (e.g., liquid biopsy), or the recommendation to select treatment with incomplete/available biomarkers. For 16.2% of patients, a liquid biopsy was favoured. 

### 3.2. Total Turnaround Time (Total TAT)

**Figure 4:**
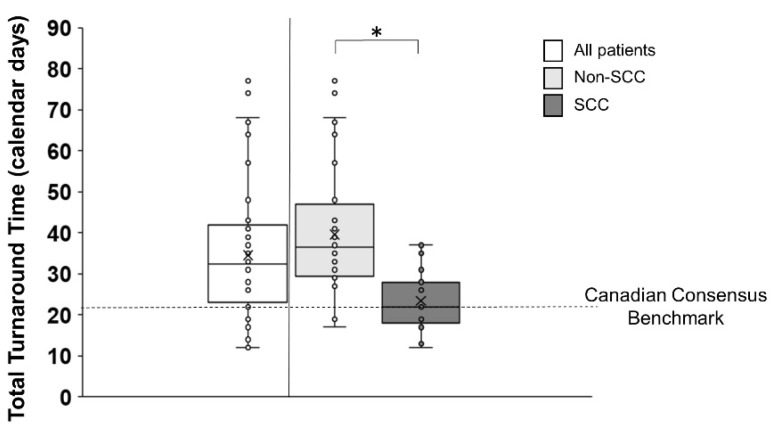
Total turnaround time (Total TAT) depicted across all patients (white bar) and divided into non-SCC (light grey bar) and SCC (dark grey bar) patients with individual participant data (small circles). The median and mean are represented by a horizontal solid line and an X in the box plot. The horizontal dashed line across the figure represents the Canadian Consensus benchmark of 21 calendar days for total TAT. The median total TAT across all patients was 32.5 (IQR 23–42) calendar days. The median total TAT for non-SCC patients was 36.5 (IQR 29.5–47) calendar days and 22 (IQR 18–28) calendar days for SCC patients. An asterisk denotes significant differences between non-SCC and SCC groups.

Out of 123 patients, total TAT was determined for 75 patients, with the remaining 48 being excluded either because of insufficient tissue (N = 9) or on the basis of no available report in the EMR (N = 39). The median total TAT across all patients was 32.5 (IQR 23–42) calendar days. When separated by histology, the median total TAT was significantly greater for non-SCC patients (36.5 (IQR 29.5–47) calendar days) than SCC patients (22 (IQR 18–28) calendar days) (z = −4.6, *p* < 0.001) (Figure 4). 

### 3.3. Pre-Laboratory Time

The median pre-laboratory time was 5 (IQR 3–8.75) calendar days measured in 111 out of 123 eligible patients (Table 4). Twelve patient data points were excluded because the biopsies were performed at another hospital. 

### 3.4. Laboratory Testing Time

**Figure 5:**
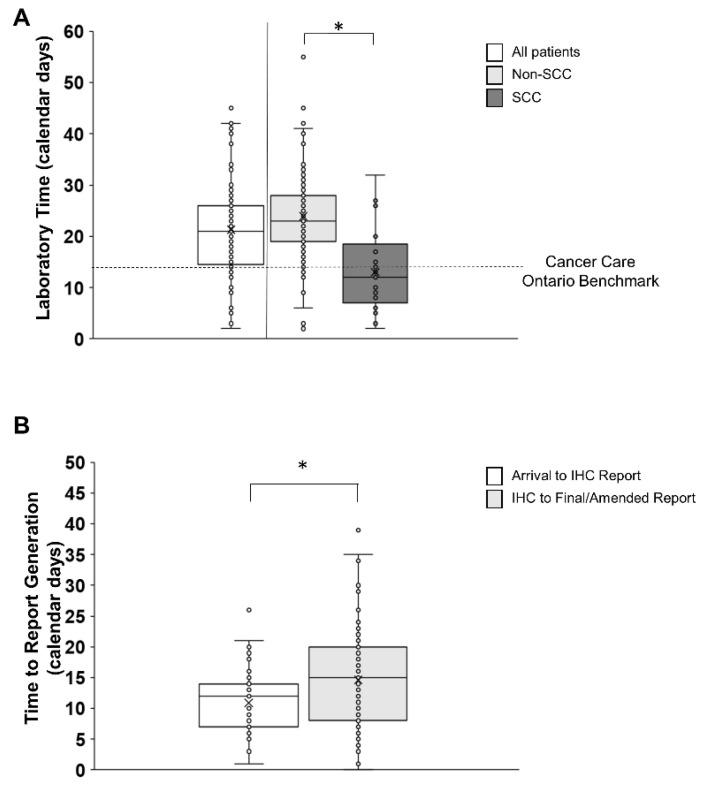
Graphs depicting laboratory time in non-SCC and SCC patients (**A**) and partial (IHC) and final (NGS) report generation in non-SCC patients (**B**). The horizontal line and X in the box plot represent the median and the mean, respectively. (**A**) Laboratory time depicted across all patients (white bar) and divided into non-SCC (light grey bar) and SCC (dark grey bar) patients with individual participant data (small circles). The median laboratory time across all patients was 21 (IQR 14–27) calendar days. The median laboratory time was longer for non-SCC than SCC patients and also longer for non-SCC patients compared to the provincial benchmark of 14 calendar days (dashed horizontal line). An asterisk denotes significant differences between non-SCC and SCC patients. (**B**) Partial IHC (white bar) and final NGS (light grey bar) report time for non-SCC patients with individual participant data (small circles). The median specimen arrival to partial IHC report generation was 12 (IQR 7–14) calendar days, while the median partial IHC report to final amended genetic report generation was 15 (IQR 8–20) calendar days. An asterisk denotes significant differences between time to IHC report generation and time to final amended genetic report generation.

The median time of laboratory testing across all patients was 21 (IQR 14–27) calendar days (Figure 5A). When considering the histological subtype, the median SCC laboratory time was 12 calendar days (IQR 7–18.5), which was below the target of 14 calendar days recommended by provincial guidelines [5]. However, the non-SCC laboratory median testing time was 23 (IQR 19–28) calendar days, which was above the provincial target of 14 calendar days (Figure 5A). Overall, only 14.6% of non-SCC patients met the tissue pathway target compared to 66.7% of SCC patients. For non-SCC patients, the median time for the laboratory receipt of a specimen to completion of the partial report was 12 (IQR 7–14) calendar days, and the median time from completion of the partial report to the final amended report was 15 (IQR 8–20) calendar days (Figure 5B). Among non-SCC samples, 23 were found to have a final report incorporating both IHC and NGS results, but not a partial report, and therefore were not included in the above calculations. 

### 3.5. Post-Laboratory Time

For non-SCC patients, the median post-laboratory time was 2 (IQR 1–6) calendar days for the partial (IHC) report (Table 4). Out of 90 eligible non-SCC patients, 23 patients had a final report only, while the partial report was not in the EMR for 6 additional patients. In the same group of patients, the final/amended report (IHC and NGS) post-laboratory time was 3 (IQR 1–8) calendar days (Table 4). In this group, 34 patients did not have their final reports in the EMR. The final report for SCC patients took a median of 3 (IQR 1–3) calendar days to be scanned into the EMR. Out of 27 SCC patients, 2 patients did not have the final report in the EMR. In order to guide treatment decisions, medical oncologists were able to track these missing reports by contacting the reference laboratory directly. Although the molecular testing had been performed/reported by the MRL, these reports were not available on the community hospital EMR for unknown reasons. 

### 3.6. Treatment, Hospitalization, and Death before Optimal Treatment Prescription

While waiting for biomarker results, 52.8% of patients had palliative radiation. This included 14.6% of patients receiving radiation to the brain. Empiric chemotherapy was prescribed in 8.1% of patients while awaiting biomarker results, with the majority of patients receiving a standard platinum doublet chemotherapy such as carboplatin and pemetrexed. Best supportive care was recommended for 16.2% of patients. While waiting for optimal treatment based on biomarker testing, 20.3% of patients were hospitalized and 7.3% died. 

### 3.7. Time to Optimal Treatment Decision (TOTD and TOTSD)

**Figure 6:**
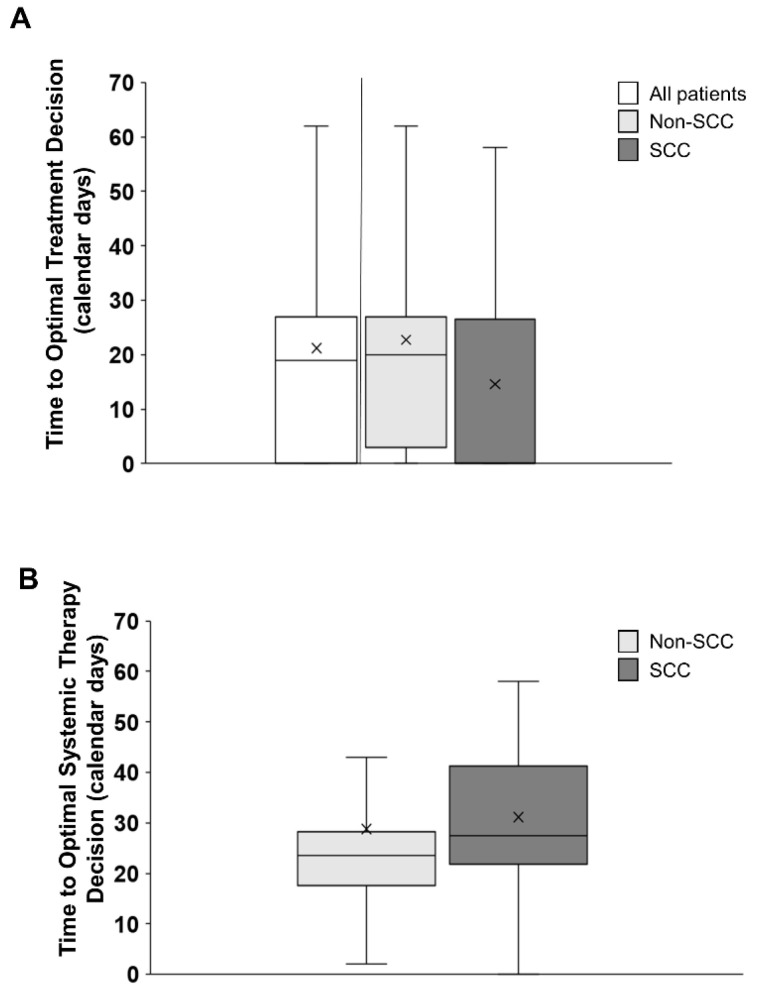
Time to optimal treatment decision (TOTD) (**A**) and time to optimal systemic therapy decision (TOTSD) (**B**). (**A**) The median TOTD across all patients was 19 (IQR 0–27) calendar days. The median TOTD was 20 (IQR 3–27) calendar days for non–SCC patients and 0 (IQR 0–26.8) calendar days for SCC patients. (**B**) The median TOTSD for non–SCC patients requiring biomarker results for optimal treatment prescription was 23.5 (IQR 17.5–28.2) calendar days and 27.5 (IQR 21.7–41.2) calendar days for SCC patients.

The median time to optimal treatment decision across all patients irrespective of histology was 19 (IQR 0–27) calendar days. The median TOTD was 20 (IQR 3–27) calendar days for non-SCC and 0 (IQR 0–26.5) calendar days for SCC patients (Figure 6A). There were no significant differences between the two groups owing to the large variability in the results (*p* = 0.08). When excluding patients who were prescribed best supportive care, and only considering patients in whom systemic therapy was being considered, the time to optimal systemic therapy decision (TOTSD) was 21.5 (IQR 10.5–28) calendar days. When evaluated by histology subtype, TOTSD was a median of 23.5 calendar days for non-SCC patients (IQR 17.5–28.2) and a median of 27.5 (IQR 21.7–41.2) calendar days for SCC patients (Figure 6B). There were no significant differences between the two patient groups (*p* = 0.21).

Optimal systemic treatment was prescribed in 58% (71 out of 123 patients). Single-agent immune checkpoint inhibitors were prescribed in 24.4% (N = 30) patients, a combination of immunotherapy and chemotherapy in 21.1% (N = 26) of patients, targeted therapy in 10.6% (N = 13) patients, and chemotherapy alone in 1.6% (N = 2) patients. Best supportive care was recommended at first consult for 20 patients (16.2%). Empiric chemotherapy was prescribed at first consult for 8.1% (10/123 patients), all of whom did not have the completion of core biomarkers. Upon the completion of biomarkers, seven had actionable mutations resulting in a change in treatment plan which occurred at a median of 31 (IQR 23.5–48) calendar days. 

## 4. Discussion

### 4.1. Delays in Pre-Laboratory, Laboratory, and Post-Laboratory Time

This is the first report to evaluate all aspects of the lung cancer tissue and treatment pathway at a large community hospital affiliated with a regional cancer centre that is dependent on off-site testing for core biomarkers in advanced NSCLC. Despite the integration of reflex testing for biomarkers, 87.9% of the patients did not have core results available at the time of referral, while 79.7% of the patients did not have core results available at the time of consultation with a medical oncologist. Without this information, optimal treatment could not be prescribed for the majority of patients when meeting their oncologist for the first time. Medical oncology consults are scheduled at a frequency that also adheres to provincial guidelines. As such, the lack of core biomarkers resulted in the need for a second, future appointment to make an optimal treatment decision. Based on the severity of symptoms, a minority of patients underwent surrogate treatments such as palliative radiation or palliative chemotherapy (52.8% and 8%, a total of approximately 61%) as temporizing measures pending personalized medicine. As shown in the statement above on switching therapy after the completion of biomarkers, a safer and more effective option such as immunotherapy or targeted therapy would have been available to them sooner if the biomarker results were available at the first consult. 

The time from the assignment of histological diagnosis of lung cancer to the receipt of the specimen at the MRL was a median of 5 calendar days. Another study where biomarker testing was conducted off-site reported a shorter pre-laboratory time of 3 business days [10]. According to the College of American Pathologists and Canadian consensus recommendations, the tissue should be received for molecular testing within 3 working days from diagnosis to avoid delays in biomarker testing [3,14]. The internal handling of tissue and variability in transportation time were cited as sources of delay in pre-laboratory time [18,19]. The delays in tissue transport to the MRL observed in this study may be related to human resource constraints in tissue preparation, budgetary differences, processing activities such as batching, and requests for additional samples. A review of internal processes should be performed to examine where avoidable delays may be occurring. 

The largest discrepancy from provincial benchmarks was seen in the laboratory phase of testing. It is worth highlighting that testing algorithms for SCC samples are different from non-SCC samples. SCC samples are tested by IHC alone for PD-L1 results, which amounts to a shorter laboratory time than non-SCC samples [9]. Non-SCC samples undergo IHC and, more importantly, NGS testing for the key biomarker EGFR, as defined in the ASCO CAP guideline [20]. Optimal therapy selection for non-SCC patients is highly dependent on the EGFR status and increasingly on the growing number of actionable mutations.

While the median laboratory time for SCC patients of 12 calendar days met the provincial benchmark of 14 days, 33.3% of SCC patients did not meet these recommendations. For the more prevalent non-SCC population, the median laboratory time was 23 days and 85.4% of patients did not meet the provincial benchmark. The critical delay in non-SCC patients was not at the level of IHC testing (median 12 calendar days), but rather at NGS testing whereby an additional 15 calendar days were required before the issue of the final/amended report. The authors were unable to definitively identify the point at which tissue was received by the NGS laboratory from the IHC reference laboratory; thus, the interpretation is that 15 calendar days is an underestimation of the true time required to complete NGS testing. 

The delay in the non-SCC laboratory phase is likely to be the consequence of samples being transported and processed at two different off-site laboratories located in two separate hospitals. Testing for non-SCC samples is carried out sequentially. IHC analysis (PD-L1, ALK) is performed first at one laboratory, after which the IHC lab staff prepare the tissue for genetic evaluation. The tissue is then transported to another laboratory for NGS (EGFR). This delay may also be due to the inherently more complex testing for NGS. Bottlenecks in testing also occur when reference laboratories servicing several hospitals are faced with increasing demand and limited resources. 

An academic center in Ontario was able to reduce the laboratory time from 8 calendar days for samples tested off-site to 3 calendar days by transitioning to on-site testing [10]. This example demonstrates how laboratory time can be reduced when one facility is designated to service one centre alone. This also suggests that transitioning testing to on-site eases the pressures faced by reference laboratories, which are responsible for processing and testing samples from multiple hospitals. 

Lastly, delays in the post-laboratory phase of testing were identified. The median post-laboratory testing time was 3 calendar days for both SCC and non-SCC samples, despite the Canadian Consensus recommendations of less than 24 h [3]. At the community hospital where this review was conducted, both the partial (IHC) and final/amended reports (IHC and NGS) were faxed to the authorizing provider at the pathology department and subsequently manually uploaded into the EMR. An unanticipated finding from this study was that out of the 123 eligible patients, 39 did not have a report available in the local EMR, representing nearly a third of all samples. Although accessing results through the EMR is the optimal pathway for medical oncologists to access laboratory results, many used an alternative electronic platform, or email/telephone communication with the reference laboratory to obtain the results of the biomarker testing. It is hypothesized that the high number of EMRs that were missing reports was due to human error since human intervention was involved at multiple points in post-laboratory testing: faxing a paper copy of the report from the reference laboratory to the local hospital, filing the report at the local hospital, and manually scanning the copy into the EMR. The automatic integration of test results through EMR may reduce post-laboratory time to meet appropriate benchmarks. This should be explored in future quality improvement studies. 

### 4.2. Delays in Optimal Treatment Decisions

Time to optimal treatment decision was not statistically significant between histological subtypes, likely owing to the large variability in results. The majority of patients were prescribed time-dilating treatments either to treat symptoms or to delay life-threatening cancer progression (52.8% and 8%, total of approximately 61%) while tumor profiling was pending. Biomarker results for the majority of these patients (70%) prompted a change to optimal targeted therapy. This highlights the impact of biomarker availability for optimal patient care, as personalized care has proven to improve outcomes compared to chemotherapy or symptom management alone [3,12,21]. Based on a previous report from a large centre, transitioning from off-site to on-site testing considerably improved the number of patients for whom all biomarkers were available at consultation. This, in turn, reduced the time to treatment decisions [10,11,15]. Faster treatment decisions have been shown to improve response rates, progression-free survival, quality of life, and, in many cases, overall survival [12]. Although not formally evaluated in this review, previous works on the psychosocial impact of delays in the cancer diagnostic period have been shown to result in patient anxiety/depression, reduced trust in the cancer team, and less treatment acceptance [22,23,24].

## 5. Limitations

The limitations of this study include the small sample size, which did not allow for the comparison of the differences in lung cancer pathways between patients who met the provincial guidelines and those who did not. However, comparisons were made between the two common NSCLC subtypes. This was a retrospective review, and, in some instances, the data were missing from the EMR system. The exact date and time the medical oncologist reviewed the biomarker results remain unknown, as these may have been accessed through multiple pathways, including the EMR system or another provincial platform affiliated with the hospital. Lastly, the time from consult to treatment decision may have been influenced by factors other than biomarker test results, including the decision to complete radiation treatment first, hospitalization, or other patient or scheduling barriers. 

## 6. Conclusions

The majority of patients with advanced NSCLC in this study did not meet provincial benchmarks for biomarker testing. A substantial number of patients did not have the biomarker results scanned in the EMR system, and this should be the focus of immediate quality improvement. The longest biomarker reporting delay was observed in patients with non-SCC histology during the laboratory phase of testing. Transitioning from off-site testing to on-site testing could significantly minimize the laboratory component of biomarker delays. According to previous reports [11], a major barrier to implementing in-house NGS testing is the cost of equipment and testing. However, adopting in-house testing significantly reduces the time to the initiation of optimal treatment and reduces the costs of patient care [25]. NGS testing is complex and requires greater resources and expertise [11] that may not be currently available in smaller centres. In the event that in-house testing is not available, immediate efforts are needed for short-term quality improvements to streamline pre- and post-laboratory workflow to improve the total biomarker testing turnaround time. The field of molecular testing is rapidly evolving and the number of therapeutic options for patients based on molecular targets is expanding. In 2023, Cancer Care Ontario updated testing guidelines to include fourteen biomarkers in patients with non-SCC histology [26]. This review affirmed the need to expand regional resources to meet the clinical needs for advanced NSCLC care in community settings.

## Figures and Tables

**Table 1:** Demographic characteristics of the NSCLC patients.

Age at Consult (Years), Mean (SD)	70.7 (9.6)
**Sex (Male), N (%)**	60 (48.8%)
**Pathology subtype, N (%)**	
Non-SCC	90 (73.1%)
SCC	27 (22%)
Other (neuroendocrine; spindle cell)	2 (1.6%)
Unknown	4 (3.3%)
**Stage, N (%)**	
Stage IIIB	12 (9.8%)
Stage IIIC	3 (2.4%)
Stage IV	108 (87.8%)
**Comorbidities, N (%)**	
No Comorbidity	55 (44.7%)
1 Comorbidity	39 (31.7%)
2 Comorbidities	21 (17.0%)
≥3 Comorbidities	8 (6.5%)
**Smoking History, N (%)**	
Smoker	99 (80.5%)
Never Smoker	24 (19.5%)
**Performance Status (ECOG), N (%)**	
Zero	1 (0.8%)
1–2	41 (33.3%)
3	16 (13.0%)
4–5	4 (3.3%)
Not stated	52 (42.3%)
Described qualitatively (“excellent”; “too poor for systemic treatment”; “bedridden”; “poor”; “borderline”;	9 (7.3%)

**Table 2:** Biomarker accession: PD-L1, ALK, and EGFR.

Outcome Measure	N (percentage)
**Liquid Biopsy Performed**	20 (16.2) (18 non-SCC, 1 other, 1 undefined)
**Insufficient tissue, repeat biopsy**	21 (17), 7
**Biomarker Results Available at Referral (all patients)**	15 (12.2)
Non-SCC	10 (11.1)
SCC	5 (18.5)
**Biomarker Results Available at Consult (all patients)**	25 (20.3)
Non-SCC	13 (14.4)
SCC	11 (40.7)
Other histology	1 (0.8)

**Table 3:** Final molecular biomarker testing results.

EGFR	All (%)	Non-SCC (%)	SCC (%)	Other (%)
Positive	13 (10.6%)	12 (13.3%)	0 (0%)	1 (16.6%)
Negative	71 (57.7%)	65 (72.2%)	3 (11.1%)	3 (50%)
Not tested	25 (20.3%)	3 (3.3%)	22 (81.5%)	0 (0%)
Insufficient Tissue	14 (11.4%)	10 (11.1%)	2 (7.4%)	2 (33.3%)
**ALK**				
Positive	1 (0.8%)	1 (1.1%)	0 (0%)	0 (0%)
Negative	92 (74.8%)	83 (92.2%)	4 (14.8%)	5 (83.3%)
Not tested	21 (17.1%)	1 (1.1%)	20 (74.1%)	0 (0%)
Insufficient Tissue	9(7.3%)	5 (5.5%)	3 (11.1%)	1 (16.6%)
**PD-L1**				
Positive (>50%)	59 (48%)	46 (51.1%)	12 (44.4%)	2 (33.3%)
Low Positive (1–49%)	24 (19.5%)	17 (18.8%)	6 (22.2%)	1 (16.6%)
Negative (<1%)	29 (23.6%)	20 (22.2%)	7 (25.9%)	2 (33.3%)
Unknown	1 (0.8%)	1 (1.1%)	0 (0%)	0 (0%)
Insufficient Tissue	10 (8.1%)	6 (6.6%)	3 (11.1%)	1 (16.6%)
**ROS-1**				
Positive	3 (2.4%)	3 (3.3%)	0 (0%)	0 (0%)
Negative	22 (17.9%)	20 (22.2%)	0 (0%)	3 (50%)
Not tested	91 (74%)	61 (67.8%)	27 (100%)	3 (50%)
Insufficient tissue	6 (4.9%)	5 (5.6%)	0 (0%)	0 (0%)
Equivocal	1 (0.8%)	1 (1.1%)	0 (0%)	0 (0%)

**Table 4:** Duration of pre-laboratory, laboratory, and post-laboratory testing.

Outcome Measure	All Patients (Median and IQR; Calendar Days)	Non-SCC (Median and IQR; Calendar Days)	SCC (Median and IQR; Calendar Days)	Consensus or Guideline
**Pre-laboratory Testing**	5 (3, 8.75)	-	-	
Histopathology to Tissue Arrival	5 (3, 8.75)			**≤3 business days**
**Laboratory Testing**	**21 (14, 27)**	**23 (19, 28)**	**12 (7, 18.5)**	**≤10 business days** **≤14 calendar days**
Time to Partial IHC Report (ALK, PD-L1)	-	12 (7, 14)	12 (7, 18.5)	
Time from Partial IHC Report to Amended/ Final Report (EGFR, etc.)	-	15 (8, 20)	N/A	
**Post-laboratory Testing**				**≤24 h**
Time to Partial IHC Report scanned in EMR	-	2 (1, 6)	3 (1, 3)	
Time to Amended/Final Report scanned in EMR	-	3 (1, 8)	N/A	

Bolded values represent the consensus or guideline timeframe, and the duration of these timeframes at this centre in comparison. IQR: interquartile range; CCO: Cancer Care Ontario; IHC: immunohistochemistry; EMR: electronic medical record.

## Data Availability

Data are available upon reasonable request from M.V.
